# Factor Xa inhibitor for venous thromboembolism management in patient with cancer: a systematic review and meta-analysis

**DOI:** 10.12688/f1000research.73883.1

**Published:** 2021-12-08

**Authors:** Johanes Nugroho Eko Putranto, Ardyan Wardhana, Yoga Alfian Noor, Pirhot Lambok Marnala Yosua Siahaan, Makhyan Jibril Al Farabi

**Affiliations:** 1 Department of Cardiology and Vascular Medicine, Faculty of Medicine, Universitas Airlangga, Surabaya, Indonesia; 2 Dr. Soetomo General Hospital, Surabaya, Indonesia; 3 Faculty of Medicine, Universitas Surabaya, Surabaya, Indonesia; 4 Department of Anesthesia and Reanimation, Faculty of Medicine, Universitas Airlangga, Surabaya, Indonesia

**Keywords:** bleeding, cancer, factor Xa inhibitor, oral anticoagulant, venous thromboembolism.

## Abstract

**Background:** An earlier systematic review reported no differences in the incidence of recurrent venous thromboembolism and major bleeding between factor Xa inhibitors and standard anticoagulation.

The present meta-analysis aimed to assess the effectiveness of factor Xa inhibitors for the management of venous thromboembolism (VTE), specifically in patients with cancer, as there were more randomized clinical trials (RCTs) available.

**Methods:** The PubMed and Cochrane Library databases were systematically screened for all RCTs assessing factor Xa inhibitor efficacy for VTE management in cancer patients. Using RevMan 5.3, we performed a Mantel–Haenszel fixed-effects meta-analysis of the following outcomes: recurrent VTE, VTE events, and major bleeding rates.

**Results:** We identified 11 studies involving 7,965 patients. Factor Xa inhibitors were superior in preventing VTE recurrence, compared to low-molecular-weight heparin (LMWH) (OR 0.60; 95% CI 0.45–0.80; P < 0.01) and vitamin K antagonists (VKA) (OR 0.51; 95% CI 0.33–0.78; P < 0.01). As prophylaxis, factor Xa inhibitors had a similar rate of VTE compared to VKAs (OR 1.08 [95% CI 0.31–3.77]; P = 0.90) and a lower rate compared to placebo (OR 0.54 [95% CI 0.35–0.81]; P < 0.01). Major bleeding rates were higher with factor Xa inhibitors than with LMWHs (OR 1.34 [95% CI 0.83–2.18]; P = 0.23), but significantly lower than VKAs (OR 0.71 [95% CI 0.55–0.92]; P < 0.01).

**Conclusions:** Factor Xa inhibitors are effective for VTE management in patients with cancer; however, they are also associated with an increased bleeding risk compared to LMWH, but decreased when compared to VKA.

## Introduction

Cancer patients are five times more likely to experience venous thromboembolism (VTE) than the general population. ^1^ Second only to cancer itself, VTE is the second most common cause of mortality in cancer patients. ^2^ According to previous clinical management recommendations, the typical VTE treatment in cancer patients involves the initial use of parenteral low-molecular-weight heparin (LMWH) followed by long-term use of oral vitamin K antagonists (VKA). ^3^ However, recent recommendations proposed factor Xa inhibitors as one of the options of the main initial treatment for VTE. ^4^

Factor Xa inhibitors are preferred over LMWH and VKA because they conveniently do not require injections every day compared to LMWH, their more predictable effects, lack of monitoring or frequent repeat doses, and fewer drug interactions compared to VKA. ^5^ An earlier systematic review reported differences between factor Xa inhibitors and standard anticoagulation drugs in the incidence of recurrent VTE and major bleeding. ^6^ Based on this research, the present meta-analysis aims to evaluate the effectiveness of factor Xa inhibitors for the management of venous thromboembolism, particularly in patients with cancer.

### Ethical considerations

Ethical approval for this research was obtained from the Dr. Soetomo General Hospital Surabaya Ethical Committee in Health Research (1964/KEPK/IV/2020).

### Trial registry

UMIN Clinical Trial Registry (UMIN ID 000040346).

## Methods

We adopted the Preferred Reporting Items for Reviews and Meta-Analyses guidelines for analysis reporting. ^7^ Any RCTs that studied VTE rates or major bleeding, as primary or secondary outcomes, in cancer patients who received an oral factor Xa inhibitor were included. Phase II trials, trials with an antiplatelet control group, and trials using an anticoagulant as VTE post-procedure prophylaxis were excluded.

We conducted a systematic search using the PubMed and Cochrane Library databases on April 24, 2020, after gaining approval from the Institutional Review Board. As for the title, abstract, and medical subject heading, we used search terms like “cancer,” “factor Xa inhibitor,” “oral anticoagulant,” “venous thromboembolism,” “apixaban,” “rivaroxaban,” “edoxaban,” “prophylaxis,” “bleeding,” “thromboembolism,” “thromboprophylaxis,” “randomized,” and “rct.”

We screened more studies by looking at the references in the included articles. Two investigators independently selected studies, with disagreements resolved through discussion and a third investigator's opinion. Thereafter, for each report, two investigators independently extracted the following information: authors, year of publication, trial name, cancer status, sample size, dose and duration of anticoagulation, duration of patient follow-up, and outcomes for the two treatment groups where available.

We determined four comparison groups: (1) factor Xa inhibitor versus LMWH as treatment for VTE; (2) factor Xa inhibitor versus VKA as treatment for VTE; (3) factor Xa inhibitor versus placebo as prophylaxis for VTE; (4) factor Xa inhibitor versus VKA as prophylaxis for VTE. The outcomes of our meta-analysis were recurrent VTE or new VTE event rates and incidence of major bleeding. VTE events were confirmed by leg vein ultrasound scanning, D-dimer testing, or both; alternatively, clinically overt pulmonary embolism was confirmed by imaging. Major bleeding was defined as in Schulman *et al.* ^8^

The Cochrane Collaboration Risk of Bias Tool was used by two independent investigators to assess the methodological quality of included studies, and the GRADE approach was employed to grade each outcome. ^9^^,^^10^ Any disputes were settled through discussion with a third investigator. We calculated odds ratios (ORs) for all outcomes at the longest follow-up period and used Review Manager ( RevMan v5.3 2014) to apply the Mantel−Haenszel fixed-effects method. We conducted a modified intention-to-treat analysis including patients who had received ≥ 1 medication dose. We planned to a conduct sensitivity analysis by removing studies likely to be biased. The I2 statistic was used to assess statistical heterogeneity between studies. If the heterogeneity was > 50%, we applied a random-effects model for analysis. ^11^

## Results

**Figure 1.**
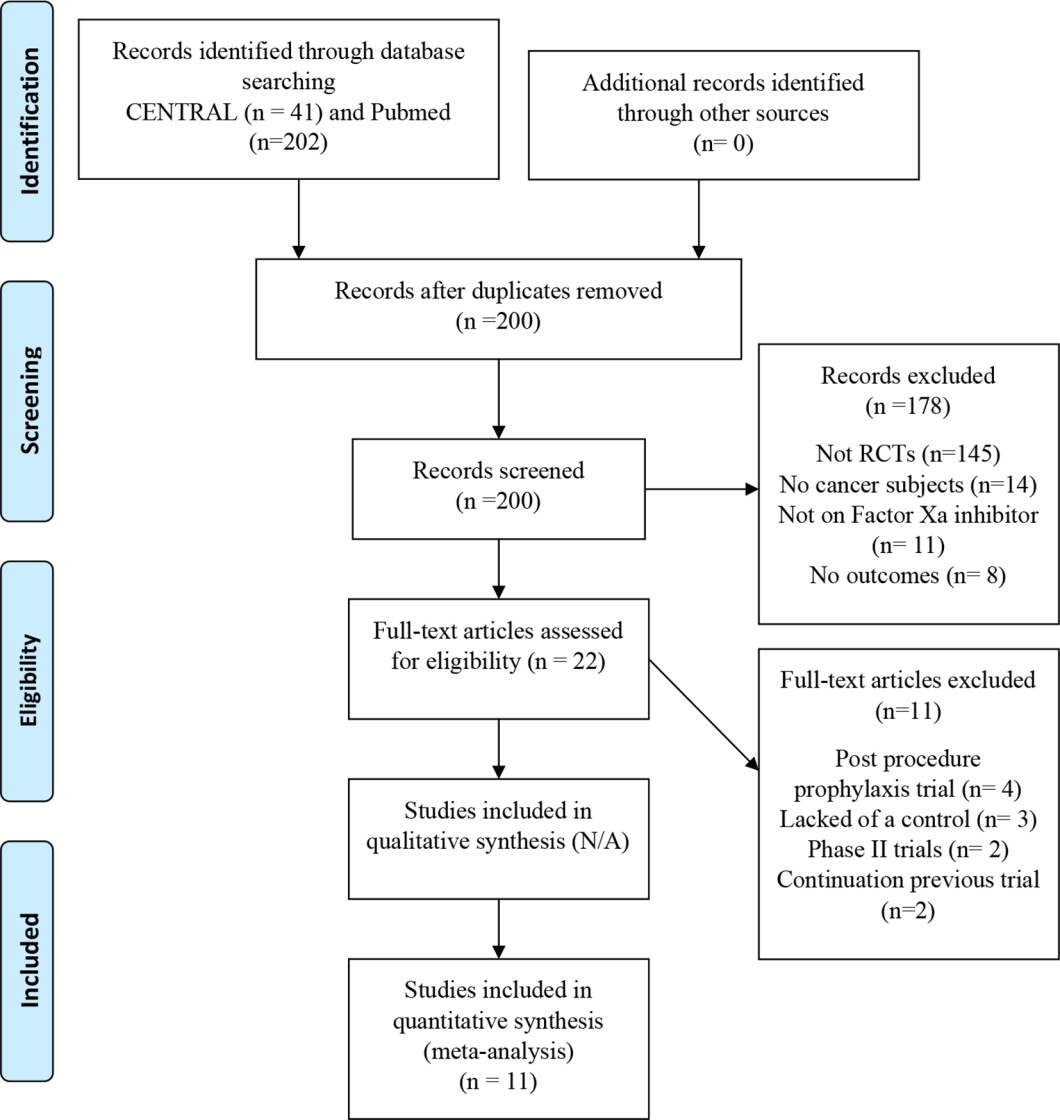
PRISMA flow diagram.

The search identified 202 citations in PubMed and 41 in the Cochrane Library, among which 43 were duplicates ( Figure 1). We found 22 more studies of which we evaluated the full text. Four studies were post-procedure prophylaxis trials, three lacked a control, two were phase II trials, and two were extensions of included trials, so 11 were omitted. As a result, we could include 11 studies in our analysis. ^12^^–^^22^

Table 1 lists the characteristics of the included studies. There were four trials on apixaban, four on rivaroxaban, and three on edoxaban. The study size ranged from 300 to 1,170 patients. Five studies were subgroup analyses of patients with cancer from larger primary trials. ^12^^–^^16^ We pooled their data only from the subgroup of patients with cancer, not all study population. One study was a pooled analysis of the subgroup of patients with cancer in “sister” trials. ^17^ Four trials ^13^^,^^18^^–^^20^ compared factor Xa inhibitors with LMWH, and three ^12^^,^^14^^,^^17^ compared factor Xa inhibitors with VKA as a VTE treatment. Two trials ^15^^,^^16^ compared factor Xa inhibitors with placebo and two ^21^^,^^22^ compared factor Xa inhibitors with VKA as prophylaxis of VTE. We included one trial that investigated two doses of edoxaban for VTE prophylaxis, where the outcomes of both groups were combined and analyzed as one intervention group. ^16^

**Table 1.** The characteristics of the included trials.

Author	Blinding to subjects	Population	Randomized patients	Intervention	Dose	Control	Follow up period	Death	Lost to follow up
Prins et al., 2013; EINSTEIN-DVT and PE	No	Cancer patients with VTE (100% active cancer)	597	Rivaroxaban	15 mg bid for 3 wk followed by 20 mg qd	Heparin/VKA	3-12 months	30% vs 35%	N/A
Agnelli et al., 2015; AMPLIFY	Yes	Cancer patients with VTE (31.6% active cancer)	534	Apixaban	10 mg bid for 7 d followed by 5 mg bid	Heparin/VKA	6 months	N/A	N/A
Raskob et al., 2016; HOKUSAI-VTE	Yes	Cancer patients with VTE (48% active cancer)	771	Edoxaban	60 mg once daily	Heparin/VKA	3-12 months	N/A	N/A
Raskop et al., 2017; HOKUSAI-VTE	No	Cancer patients with VTE (97.9% active cancer)	1050	Edoxaban	60 mg once daily	Dalteparin (200 UI/kg/d during 30 days, then 150 UI/kg/d)	12 months	39% vs 36%	0.8% (3 vs 5)
Young et al., 2017; SELECT-D	No	Cancer patients with VTE (100% active cancer)	406	Rivaroxaban	15 mg bid for 3 wk followed by 20 mg qd	Dalteparin (200 UI/kg/d during 30 days, then 150 UI/kg/d)	6 months	75% vs 70%	0.2% (0 vs 1)
McBane et al., 2018; ADAM VTE	No	Cancer patients with VTE (100% active cancer)	300	Apixaban	10 mg bid for 7 d followed by 5 mg bid	Dalteparin (200 UI/kg/d during 30 days, then 150 UI/kg/d)	6 months	15% vs 10%	5.6% (9 vs 7)
Fanola et al., 2018; ENGAGE AF-TIMI	No	Cancer patients with AF (100% active cancer)	1153	Edoxaban	60 mg once daily or 30 mg once daily	VKA	> 2 years	32% vs 30%	N/A
Chen et al., 2019; ROCKET AF	No	Cancer patients with AF (7.8% active cancer)	640	Rivaroxaban	20 mg qd	VKA	2 years	10% vs 15%	N/A
Carrier et al., 2019; AVERT	Yes	Ambulatory patients with risk of VTE	574	Apixaban	2.5 mg bid	Placebo	6 months	12% vs 10%	4.3% (13 vs 11)
Khorana et al., 2019; CASSINI	Yes	Ambulatory patients with risk of VTE	841	Rivaroxaban	10 mg qd	Placebo	6 months	20% vs 25%	N/A
Agnelli et al., 2020; CARAVAGGIO	No	Cancer patients with VTE (97.3% active cancer)	1170	Apixaban	10 mg bid for 7 d followed by 5 mg bid	Dalteparin (200 UI/kg/d during 30 days, then 150 UI/kg/d)	6 months	23% vs 25%	1.7% (12 vs 8)

**Figure 2.**
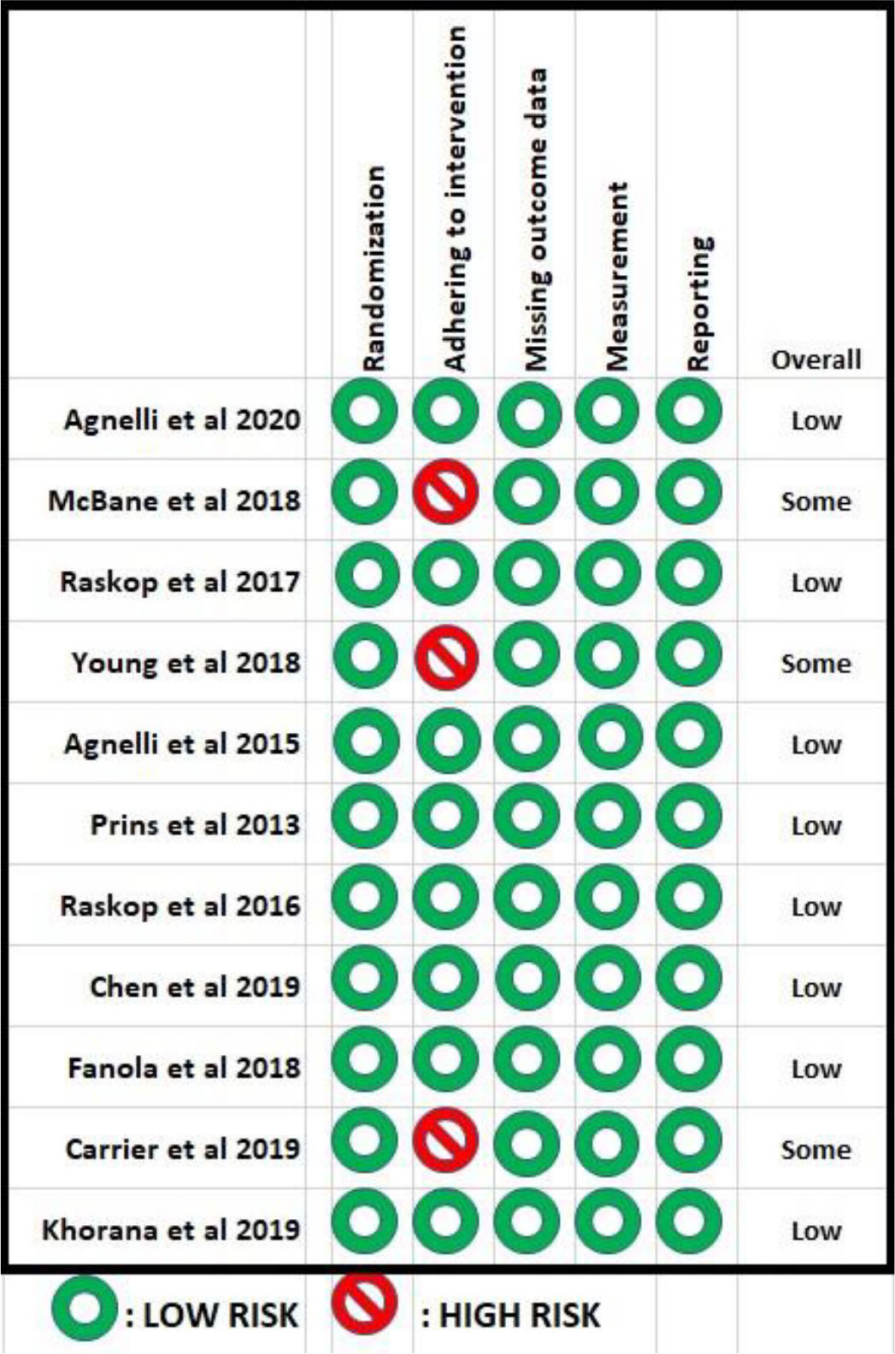
Risk of bias assessment.

The risk of bias across domains is presented in Figure 2. In most studies, the randomization process, adherence to the intervention, assessment, missing outcome results, and reporting were deemed adequate. In four trials, participants were blinded. The percentage of patients not followed up ranged from 0.2% to 5.6%. All trials reported the results from modified intention-to-treat analysis.

**Figure 3.**
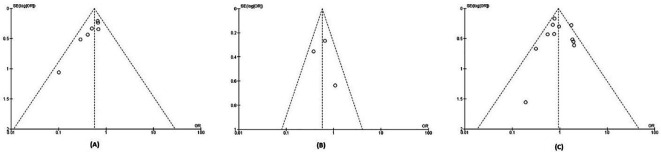
Funnel plot of (A) recurrent VTE outcome; (B) new VTE outcome; (C) major bleeding outcome.

The quality of evidence for each outcome analyzed using the GRADE approach is presented in Table 2. We did not downgrade from the risk of bias, inconsistency, indirectness, and imprecision aspect of all outcomes, because of a low risk of bias, no substantial heterogeneity, a large enough sample size, and narrow confidence interval (CI). We downgraded one level for the major bleeding outcome because the funnel plot of major bleeding outcome suggested publication bias ( Figure 3).

**Table 2.** Summary of findings.

	No of studies	Total participants	Pooled OR (95% CI)	P	I2 (P)	GRADE
Recurrence						High
vs LMWH	4	2890	0.60 (0.45, 0.80)	0.0004	26% (0.26)	
vs VKA	3	1881	0.51 (0.33, 0.78)	0.002	0% (0.37)	
New VTE						High
vs VKA	1	684	1.08 (0.31, 3.77)	0.90	N/A	
vs Placebo	2	1372	0.54 (0.35, 0.81)	0.003	31% (0.23)	
Major bleeding						Moderate
vs LMWH	4	2890	1.34 (0.83, 2.18)	0.23	28% (0.25)	
vs VKA	5	3703	0.71 (0.55, 0.92)	0.009	0% (0.72)	
vs Placebo	2	1372	1.98 (0.88, 4.44)	0.10	0% (0.96)	

**Figure 4.**
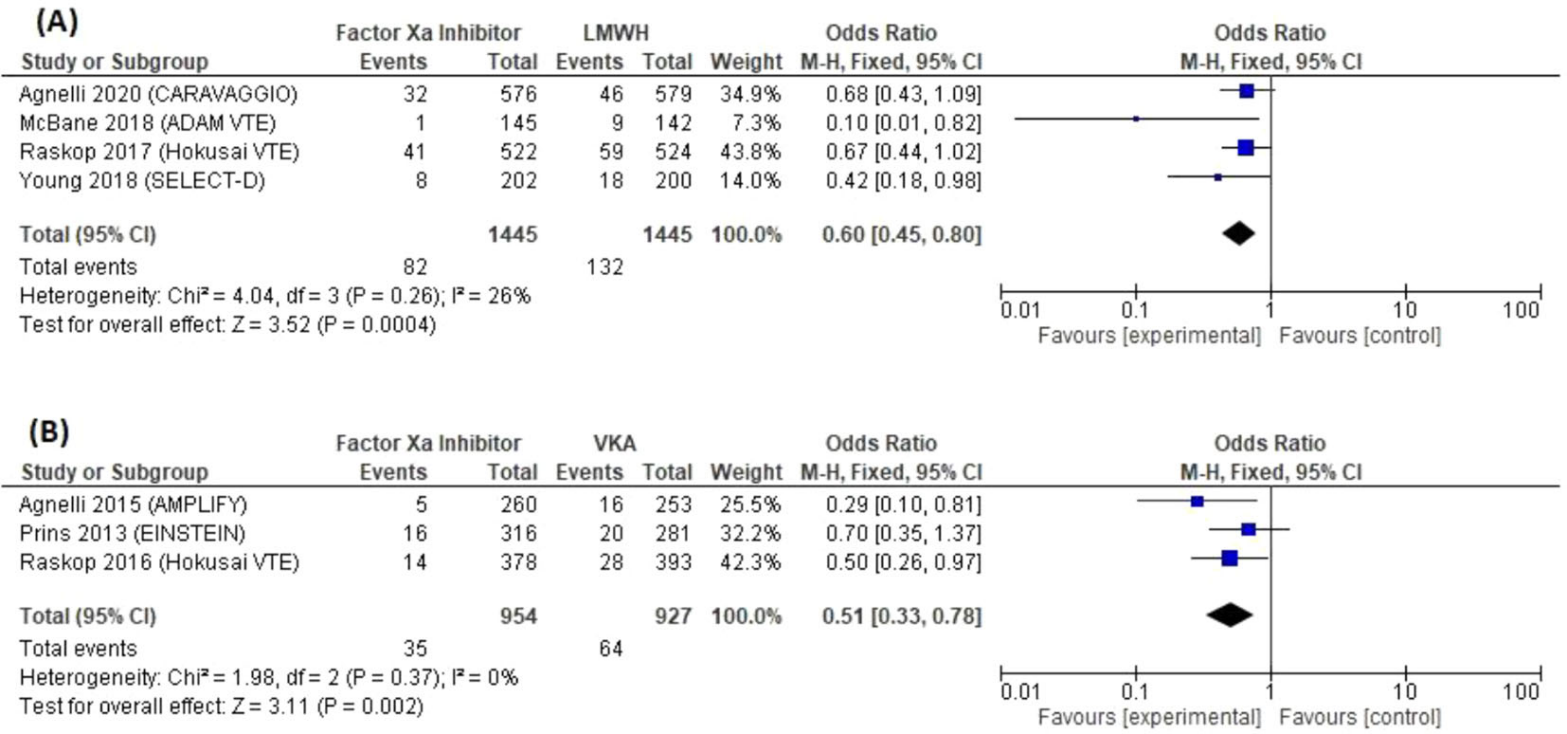
Forest plot of recurrent VTE outcome.

Seven studies involving 4,771 patients reported VTE recurrence ( Table 2). Recurrence occurred in 4.9% (117/2,399) of patients allocated to factor Xa inhibitors, 9.1% (132/1,445) allocated to LMWHs, and 6.9% (64/927) of those allocated to VKAs. In comparison ( Figure 4), the reduction of the risk of VTE recurrence with factor Xa inhibitors compared to LMWH was acceptable (four trials; OR 0.60; 95% CI 0.45–0.80; P < 0.01), without substantial heterogeneity (I2 = 26%; P = 0.26). VTE recurrence rates were lower in patients treated with factor Xa inhibitors compared to patients treated using VKAs (three trials; OR 0.51; 95% CI 0.33–0.78; P < 0.01), without substantial heterogeneity (I2 = 0%; P = 0.37).

**Figure 5.**
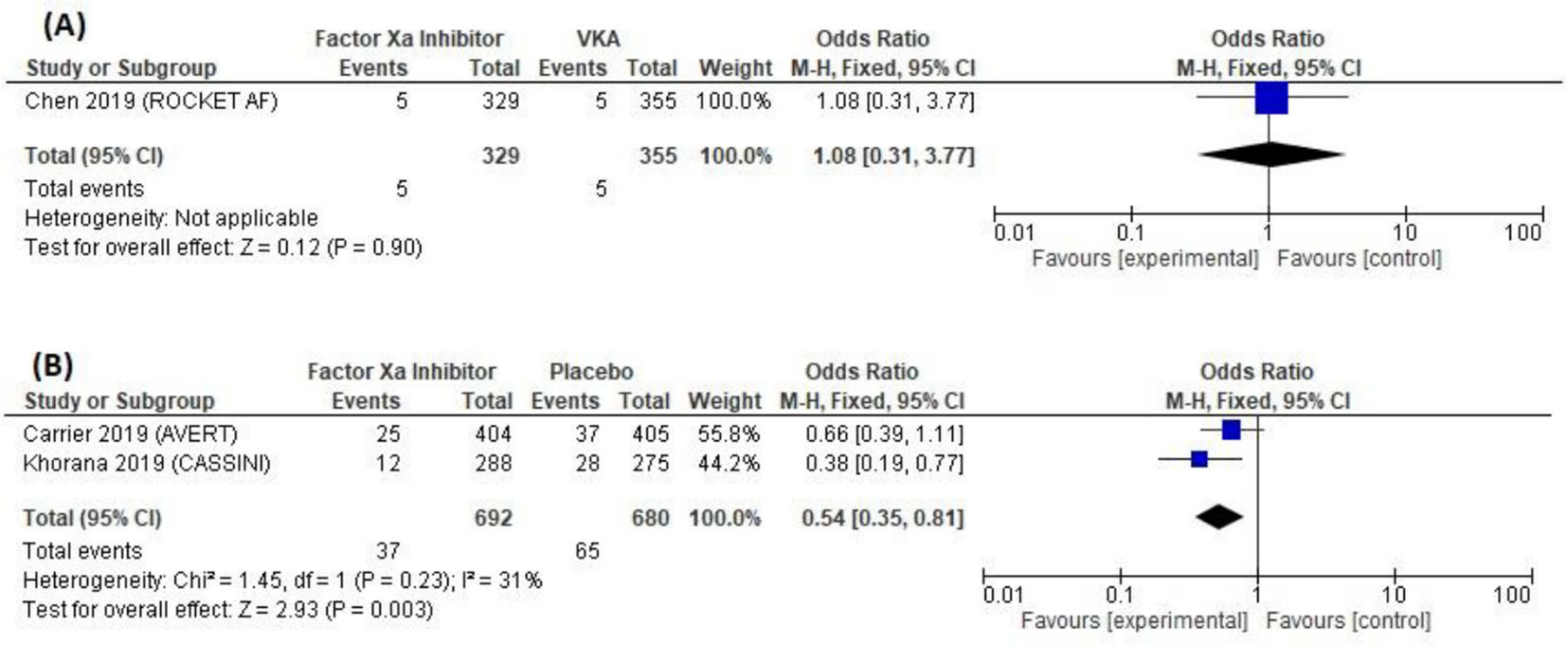
Forest plot of new VTE outcome.

Three studies, including 2,056 patients, reported the incidence of new VTE after anticoagulant prophylaxis. The factor Xa inhibitor group had a 4.1% (42/1,021) VTE occurrence rate, while the VKA and placebo groups each had 1.45% (5/355) and 9.6% (65/680), respectively. According to the meta-analysis shown in Figure 5, there were similar VTE incidences in the factor Xa inhibitor and the VKA groups (one trial; OR = 1.08 [95% CI, 0.31–3.77]; P = 0.90); however, the heterogeneity analysis could not be applied. The estimated effect of factor Xa inhibitors on VTE incidence compared to placebo showed a statistically significant reduction (two trials; OR = 0.54 [95% CI, 0.35–0.81]; P < 0.01), without substantial heterogeneity (I2 = 31%; P = 0.23).

**Figure 6.**
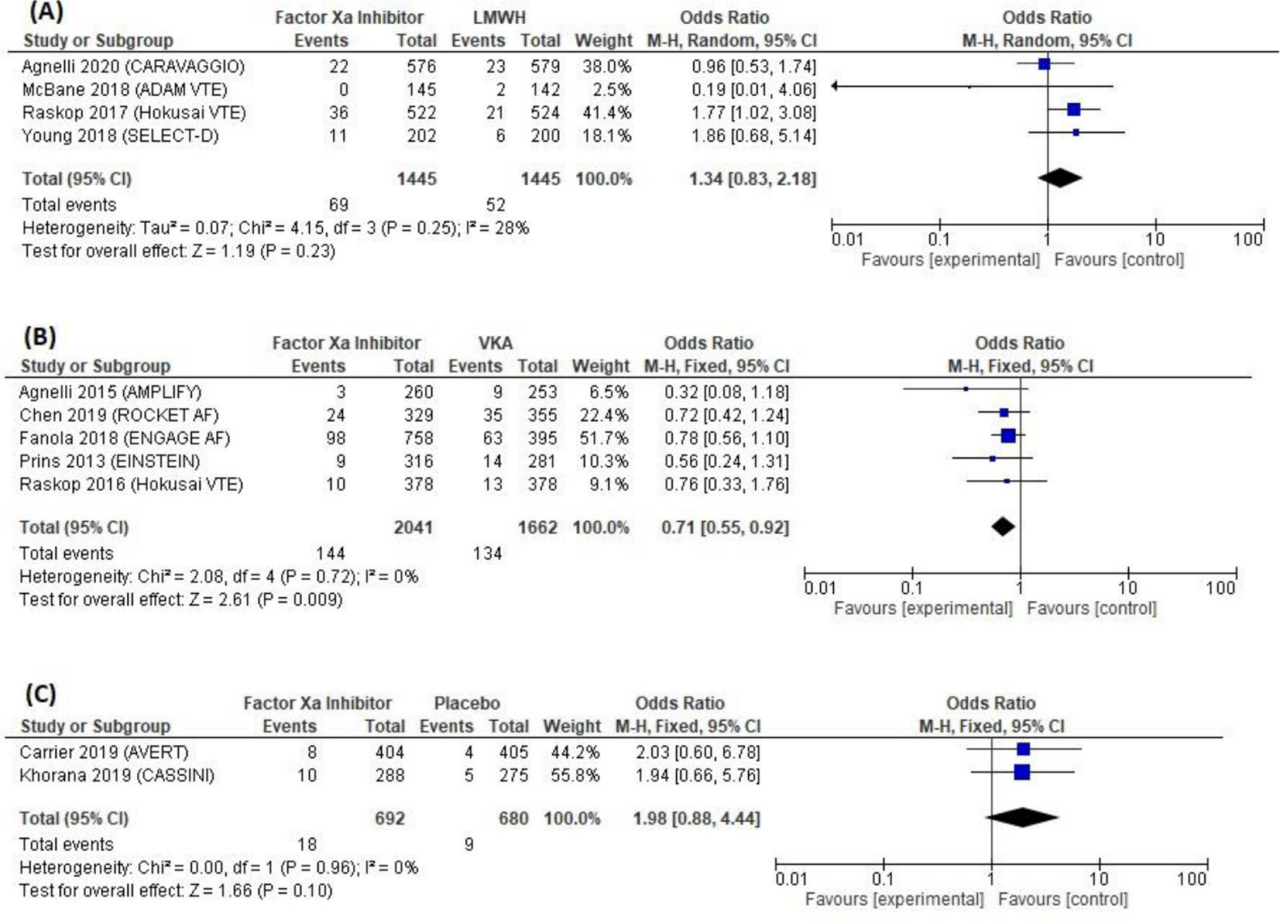
Forest plot of major bleeding outcome.

Eleven studies, including 7,965 patients, reported major bleeding ( Table 2). Major bleeding occurred in 5.5% (231/4,178) of patients allocated to factor Xa inhibitors, 3.6% (52/1445) to LMWHs, 8.1% (134/1,662) to VKAs and 1.3% (9/680) to placebo. According to the meta-analysis shown in Figure 6, the acceptable increase of risk cannot be confirmed from the description of major bleeding with factor Xa inhibitors compared to LMWH, as based on an OR of 1.34 (95% CI, 0.83–2.18) with a P = 0.23, which is not statistically significant. However, factor Xa inhibitors significantly reduced the risk of major bleeding compared to VKAs (five trials; OR = 0.71 [95% CI, 0.55–0.92]; P = 0.009), without substantial heterogeneity (I2 = 0%; P = 0.72). The risk of major bleeding was higher with factor Xa inhibitors versus placebo (two trials; OR = 1.98 [95% CI, 0.88–4.44]; P = 0.10) but not statistically significant, without substantial heterogeneity (I2 = 0%; P = 0.96).

## Discussion

The aim of this meta-analysis was to determine the efficacy and safety of factor Xa inhibitors for VTE treatment in cancer patients. Recurrence was 4.9%, 9.1%, and 6.9 % for the factor Xa inhibitor, LMWH, and VKA groups, respectively. All were lower than the findings of a retrospective cohort study which reported an incidence of 13.1%, 17.6%, and 17.9%, respectively. ^23^ Our review of four studies involving over 4,771 patients found that factor Xa inhibitors were associated with a lower risk of VTE recurrence when compared to LMWH, and even lower when compared to VKA. This result was consistent with a recent meta-analysis which combined data from RCTs and retrospective cohort studies. ^24^

Another finding in our meta-analysis in terms of safety profiles was that factor Xa inhibitors were associated with an increased risk of bleeding when compared to LMWH, but a lower risk when compared to VKA. This result is in line with the findings of other systematic reviews. ^24^^–^^26^ However, another meta-analysis found a significantly higher incidence of bleeding (two trials, OR= 2.72 [95% CI: 1.05–7.01]; P= 0.039) with factor Xa inhibitors, relative to LMWH. ^27^ Importantly, the bleeding outcome in comparison to LMWH was the result of pooled data from nonspecific cancer patients. The results of the analysis of major bleeding in comparison to LMWH were mainly influenced by those of the HOKUSAI VTE Cancer trial and the recent CARAVAGGIO trial. ^28^^,^^29^ Both had different results: the former showed significantly higher bleeding in the edoxaban group while the second showed similar major bleeding events between groups.

Our meta-analysis also provided information about the efficacy of factor Xa inhibitors as prophylaxis, which suggested that, compared to placebo, it can significantly reduce VTE incidence. According to a recent clinical practice guideline, high-risk cancer outpatients can receive thromboprophylaxis with a factor Xa inhibitor or LMWH, in the absence of major risk factors for bleeding. ^30^ The high cost and the pain of daily LMWH injections was avoided with the factor Xa inhibitor regimen.

With respect to factor Xa inhibitors and LMWH, the inclusion of the CARAVAGGIO trial, with highly rigorous evidence, increased the accuracy of the estimated outcomes. There are a number of limitations to the current meta-analysis: the majority of the data corresponded to subgroup or post-hoc analyses. Further, the following variables were not controlled for: cancer stage, type of cancer, follow-up period. While most of the included studies evaluated patients for six months, the optimal duration of anticoagulation treatment was not evaluated to achieve an agreement. Finally, despite our systematic electronic database search and our investigation of the references in the included studies, we may have missed relevant studies.

## Conclusion

Factor Xa inhibitors are effective for VTE management in patients with cancer; however, they are also associated with an increased bleeding risk compared to LMWH, but decreased when compared to VKA.

### Data availability statement

#### Underlying data

All data underlying the results are available as part of the article and no additional source data are required.

#### Reporting guidelines ^8^

Figshare: PRISMA checklist for ‘Factor Xa inhibitor for venous thromboembolism management in Patients with cancer: a systematic review and meta-analysis’. https://doi.org/10.6084/m9.figshare.16590086.v3 ^31^

Data are available under the terms of the Creative Commons Attribution 4.0 International license (CC-BY 4.0).

## References

[1] Elyamany G Alzahrani AM Bukhary E : Cancer-associated thrombosis: an overview. *Clin Med Insights Oncol.* 2014;8:129–137. 10.4137/CMO.S18991 25520567 PMC4259501

[2] Khorana AA Francis CW Culakova E : Thromboembolism is a leading cause of death in cancer patients receiving outpatient chemotherapy. *J. Thromb. Haemost.* 2007;5:632–634. 10.1111/j.1538-7836.2007.02374.x 17319909

[3] Kearon C Akl EA Comerota AJ : Antithrombotic therapy for VTE disease: antithrombotic therapy and prevention of thrombosis, 9th ed: American College of Chest Physicians Evidence-Based Clinical Practice Guidelines. *Chest.* 2012;141:e419S–e496S. 10.1378/chest.11-2301 22315268 PMC3278049

[4] Lyman GH Carrier M Ay C : American Society of Hematology 2021 guidelines for management of venous thromboembolism: prevention and treatment in patients with cancer. *Blood Adv.* 2021;5(4):927–974. 10.1182/bloodadvances.2020003442 33570602 PMC7903232

[5] Fox BD Kahn SR Langleben D : Efficacy and safety of novel oral anticoagulants for treatment of acute venous thromboembolism: direct and adjusted indirect meta-analysis of randomised controlled trials. *BMJ.* 2012;345:e7498. 10.1136/bmj.e7498 23150473 PMC3496553

[6] Robertson L Kesteven P McCaslin JE : Oral direct thrombin inhibitors or oral factor Xa inhibitors for the treatment of pulmonary embolism. *Cochrane Database Syst. Rev.* 2015;2016(Issue12): Art. No.:CD010957. 10.1002/14651858.CD010957.pub2 PMC6463831 26636644

[7] Moher D Liberati A Tetzlaff J : The PRISMA Group. Preferred reporting items for systematic reviews and meta-analyses: The PRISMA statement. *Open Med.* 2009;3:123–130. PMC3090117 21603045

[8] Schulman S Kearon C : Definition of major bleeding in clinical investigations of antihemostatic medicinal products in non-surgical patients. *J. Thromb. Haemost.* 2005;3:692–694. 10.1111/j.1538-7836.2005.01204.x 15842354

[9] Sterne JAC Savović J Page MJ : RoB 2: a revised tool for assessing risk of bias in randomised trials. *BMJ.* 2019 Aug 28;366:l4898. 10.1136/bmj.l4898 31462531

[10] Ryan R Hill S : *How to GRADE the quality of the evidence Version 3.0.* Cochrane Consumers and Communication Group;2018 Dec [cited 2019 July 13];[about 25 p.]. Reference Source

[11] Higgins JPT Green S : *Cochrane Handbook for Systematic Reviews of Interventions Version 5.1.0.* The Cochrane Collaboration;2011 [cited 2019 July 10]. Reference Source

[12] Raskob GE Es N van Segers A : Edoxaban for venous thromboembolism in patients with cancer: results from a non-inferiority subgroup analysis of the Hokusai-VTE randomised, double-blind, double-dummy trial. *Lancet Haematol.* 2016;3:e379–e387. 10.1016/S2352-3026(16)30057-6 27476789

[13] Raskob GE Es N van Verhamme P : on behalf of the Hokusai VTE Cancer Investigators. Edoxaban for the treatment of cancer-associated venous thromboembolism. *N. Engl. J. Med.* 2018;378(7):615–624. 10.1056/NEJMoa1711948 29231094

[14] Agnelli G Buller HR Cohen A : Oral apixaban for the treatment of venous thromboembolism in cancer patients: Results from the AMPLIFY trial. *J. Thromb. Haemost.* 2015;13(12):2187–2191. 10.1111/jth.13153 26407753

[15] Chen ST Hellkamp AS Becker RC : Efficacy and safety of rivaroxaban vs. warfarin in patients with non-valvular atrial fibrillation and a history of cancer: observations from ROCKET AF. *Eur Heart J Qual Care Clin Outcomes.* 2019;5(2):145–152. 10.1093/ehjqcco/qcy040 30219887

[16] Fanola CL Ruff CT Murphy SA : Efficacy and safety of edoxaban in patients with active malignancy and atrial fibrillation: analysis of the ENGAGE AF - TIMI 48 trial. *J Am Heart Assoc.* 2018;7(16):e008987. 10.1161/JAHA.118.008987 30369307 PMC6201390

[17] Prins MH Lensing AW Bauersachs R : Oral rivaroxaban versus standard therapy for the treatment of symptomatic venous thromboembolism: a pooled analysis of the EINSTEIN-DVT and PE randomized studies. *Thromb J.* 2013;11(1):21. 10.1186/1477-9560-11-21 24053656 PMC3850944

[18] Agnelli G Becattini C Meyer G : Apixaban for the treatment of venous thromboembolism associated with cancer. *N Engl J Med.* 2020;382:1599–1607. 10.1056/NEJMoa1915103 32223112

[19] Young AM Marshall A Thirlwall J : Comparison of an oral factor Xa inhibitor with low molecular weight heparin in patients with cancer with venous thromboembolism: Results of a randomized trial (SELECT-D). *J Clin Oncol.* 2018;36(20):2017–2023. 10.1200/JCO.2018.78.8034 29746227

[20] McBane RD 2nd Wysokinski WE Le-Rademacher JG : Apixaban, dalteparin, in active cancer associated venous thromboembolism, the ADAM VTE trial. *Blood.* 2020;18(2):411–421. 10.1111/jth.14662 31630479

[21] Khorana AA Soff GA Kakkar AK : Rivaroxaban for thromboprophylaxis in high-risk ambulatory patients with cancer. *N Engl J Med.* 2019;380:720–728. 10.1056/NEJMoa1814630 30786186

[22] Carrier M Abou-Nassar K Mallick R Apixaban to prevent venous thromboembolism in patients with cancer. *N Engl J Med.* 2019;380:711–19. 10.1056/NEJMoa1814468 30511879

[23] Streiff MB Mccrae K Yannicelli D : Effectiveness and safety of anticoagulants for the treatment of venous thromboembolism in patients with cancer. *Am J Hematol.* 2018;93(5):664–671. 10.1002/ajh.25059 29396864 PMC5947542

[24] Yang M Li J Sun R : Comparison between direct factor Xa inhibitors and low-molecular-weight heparin for efficacy and safety in the treatment of cancer-associated venous thromboembolism: A meta-analysis. *J Can Res Ther.* 2019;15:1541–1546. 10.4103/jcrt.JCRT_68_19 31939435

[25] Fuentes HE McBane RD 2nd Wysokinski WE : Direct Oral Factor Xa Inhibitors for the Treatment of Acute Cancer-Associated Venous Thromboembolism: A Systematic Review and Network Meta-analysis. *Mayo Clin Proc.* 2019;94(12):2444–2454. 10.1016/j.mayocp.2019.05.035 31685262

[26] Li A Garcia DA Lyman GH : Direct oral anticoagulant (DOAC) versus low-molecular-weight heparin (LMWH) for treatment of cancer associated thrombosis (CAT): A systematic review and meta-analysis. *Thromb Res.* 2019;173:158–163. 10.1016/j.thromres.2018.02.144 29506866 PMC6119655

[27] Brunetti ND Gesuete E De Gennaro L : Direct oral anti-coagulants compared with vitamin-K inhibitors and low-molecular-weight-heparin for the prevention of venous thromboembolism in patients with cancer: A meta-analysis study. *Int J Cardiol.* 2017;230:214–221. 10.1016/j.ijcard.2016.12.168 28062137

[28] Hokusai VTE Cancer Investigators: Edoxaban for the Treatment of Cancer-Associated Venous Thromboembolism. *N Engl J Med.* 2018;378:673–674. 10.1056/NEJMe1800041 29231094

[29] Caravaggio Investigators: Apixaban for the Treatment of Venous Thromboembolism Associated with Cancer. *N Engl J Med.* 2020;382:1599–1607. 10.1056/NEJMoa1915103 32223112

[30] Key NS Khorana AA Kuderer NM : Venous thromboembolism prophylaxis and treatment in patients with cancer: ASCO clinical practice guideline update. *J Clin Oncol.* 2020;38:496–520. 10.1200/JCO.19.01461 31381464

[31] Al Farabi Jibril M : PRISMA XA Checklist and Diagram. figshare. *Dataset.* 2021. 10.6084/m9.figshare.16590086.v3

