# Supplementary material for: Factor Xa inhibitor for venous thromboembolism management in patient with cancer: a systematic review and meta-analysis
Source: F1000Res. 2021 Dec 8;10:1257. [Version 1] doi: 10.12688/f1000research.73883.1 (PMC8802150; doi:10.12688/f1000research.73883.1)
Supplement: Peer review history

# Peer review: Reviewer response for version 1

**DOI:** 10.5256/f1000research.77572.r102367

**Published:** 2022-01-28

**Authors:** Irene Terrenato

**Affiliations:** 1 Biostatistic Unit - Scientific Direction, IRCCS Regina Elena National Cancer Institute, Rome, Italy

In this study authors conducted a systematic review and a meta-analysis to explore the effectiveness of Factor Xa inhibitors compared to standard anticoagulation treatments for the management of venous thromboembolism (VTE) in patients with cancer. The study is well conducted and the methods section is complete and detailed. All the statistical analysis were properly applied and the conclusions are adequately supported by the results presented in the paper. This reviewer recognizes the value of the manuscript however minor revisions are needed to improve the paper.

 In particular:

1. **Abstract:**
  The major bleeding result should be re-written according to the text of the paper. The comparison between Factor Xa inhibitors and LMWH is not statistically significant therefore authors cannot comment and generalize it as if it were.
2. **Table 2: Please make all labels in the table more readable:**
  - Add “Xa inhibitors” in the first column for each comparison for each outcome.
  - Add “p-value” instead of “P”.
  - Add “ I ^2^ (p-value)” instead “I2 (P)”
3. **Figure 4,5, and 6:**
  In the titles, add the labels to each panel for each comparison.

Are the rationale for, and objectives of, the Systematic Review clearly stated?

Yes

Is the statistical analysis and its interpretation appropriate?

Yes

Are sufficient details of the methods and analysis provided to allow replication by others?

Yes

Are the conclusions drawn adequately supported by the results presented in the review?

Yes

Reviewer Expertise:

cancer, biostatistics

I confirm that I have read this submission and believe that I have an appropriate level of expertise to confirm that it is of an acceptable scientific standard.

# Peer review: Reviewer response for version 1

**DOI:** 10.5256/f1000research.77572.r102373

**Published:** 2022-01-28

**Authors:** Suko Adiarto

**Affiliations:** 1 Department of Cardiology and Vascular Medicine, Universitas Indonesia/ Harapan Kita National Cardiovascular Center, Jakarta, Indonesia

The reviewer, while appreciating the authors' objective, namely to provide a meta-analysis of recent studies using factor Xa inhibitors, low-molecular-weight heparin (LMWH), and vitamin K antagonists (VKA) in patients with venous thromboembolism (VTE) and cancer, intends to submit some general and specific observations:

1. The topic is an extremely important one, and the authors of this paper by accessing multiple data sources have a unique opportunity to produce a clinically important paper.
2. In results section, the authors acknowledge that all patients of interest had cancer, thus, what is of particular interest to the clinicians is while acknowledging the factor X inhibitors work, which factor X and at what dose was the most effective and or the safest, so that the clinicians can make an important decision regarding patient management. Also, the type of cancer in the included studies need to be specified.
3. In result section, the authors make an important statement that the factor Xa inhibitors are more effective than low molecular wave heparin or vitamin K antagonists. This is an important point, however the clinician requires more specific information.
4. Moreover, among the bibliographic entries only some of the meta-analytical sector studies already present in the literature are reported.

Are the rationale for, and objectives of, the Systematic Review clearly stated?

Yes

Is the statistical analysis and its interpretation appropriate?

Yes

Are sufficient details of the methods and analysis provided to allow replication by others?

Yes

Are the conclusions drawn adequately supported by the results presented in the review?

Yes

Reviewer Expertise:

Cardiovascular Medicine and Intervention

I confirm that I have read this submission and believe that I have an appropriate level of expertise to confirm that it is of an acceptable scientific standard.
